# Urine N-terminal pro-B-type natriuretic peptide and plasma proenkephalin are promising biomarkers for early diagnosis of cardiorenal syndrome type 1 in acute decompensated heart failure: a prospective, double-center, observational study in real-world

**DOI:** 10.1080/0886022X.2022.2114367

**Published:** 2022-08-24

**Authors:** Hong-Liang Zhao, Hai-Juan Hu, Xiu-Jie Zhao, Wei-Wei Chi, De-Min Liu, Qian Wang, Wei Cui

**Affiliations:** a Department of Cardiology, The Second Hospital of Hebei Medical University, Shijiazhuang, PR China; b Department of Cardiology, The First Hospital of Hebei Medical University, Shijiazhuang, PR China; c Biobank, The First Hospital of Hebei Medical University, Shijiazhuang, PR China

**Keywords:** Cardiorenal syndrome type 1, urine N-terminal pro-B-type natriuretic peptide, proenkephalin, acute decompensated heart failure, prediction

## Abstract

**Background:**

Patients with acute decompensated heart failure (ADHF) show cardiorenal syndrome type 1 (CRS-1) are more likely to have a poor outcome. However, the current criteria often lead to delayed CRS-1 diagnosis. Therefore, we evaluated the predictive value of plasma proenkephalin (pPENK) and urine NT-proBNP (uNT-proBNP) for early diagnosis of CRS-1 and vulnerable-phase prognosis in ADHF patients.

**Methods:**

The plasma NT-proBNP (pNT-proBNP), pPENK, and uNT-proBNP were measured in 121 ADHF patients on admission. The plasma neutrophil gelatinase-associated lipocalin (pNGAL) was chosen as the reference. Logistic regression was used to determine the predictors of CRS-1. The area under the receiver operating curves (ROCs) was calculated to assess the early diagnostic value of pNGAL, pPENK, and uNT-proBNP/uCr for CRS-1. To evaluate the prognostic risk of factors for the 90-d outcomes of all ADHF patients, the Cox regression was performed and the cumulative risk curve was plotted.

**Results:**

We found that pPENK [OR 1.093 (95% CI 1.022–1.169), *p* = 0.010; AUROC = 0.899 (95% CI 0.831–0.946)] and uNT-proBNP/uCr ratio [OR 1.015 (95% CI 1.003–1.028), *p* = 0.012; AUROC = 0.934 (95% CI 0.874–0.971)] could independently predict the occurrence of CRS-1 in hospitalized patients with ADHF. The pPENK [HR 1.014 (95% CI 1.000–1.042), *p* = 0.044] and uNT-proBNP/uCr ration [HR 0.998 (95% CI 0.997–1.000), *p* = 0.045] were also independent predictors of the risk of HF readmission or all-cause death 90 d after discharge in ADHF patients.

**Conclusions:**

The newly found pPENK and noninvasive test of uNT-proBNP/uCr ratio (pg/nmol) on admission may be two promising novel predictive biomarkers for early diagnosis of CRS-1 occurrence and vulnerable-phase outcomes in ADHF patients.

## Introduction

Cardiorenal syndrome type 1 (CRS-1) is characterized as a rapid worsening of cardiac function followed by acute kidney injury (AKI), which is common in patients with acute decompensated heart failure (ADHF) [1]. CRS-1 occurs in between 25 and 33% of patients admitted with ADHF [2] and is associated with a higher risk of poor outcomes [3]. The period of 90 d after hospitalization of patients with heart failure (HF) has been termed the ‘vulnerable phase’ with a poor prognosis [4]. Therefore, early diagnosis of CRS-1 in ADHF patients is crucial for formulating a treatment strategy and assessing prognosis. However, the current criteria often result in delayed diagnosis of AKI due to the measurement of serum creatinine (sCr) or urine volume according to the Kidney Disease Improving Global Outcomes (KDIGOs) [5]. Although certain biomarkers have recently been developed to help predict CRS-1 in ADHF, most of them are still not widely used in clinical practice and some findings remain controversial [6–10]. More importantly, using a single biomarker to describe the complex pathophysiology of CRS-1 is challenging and cannot reflect the clinical and pathophysiological complexity of the syndrome.

Amino-terminal pro-B-type natriuretic peptide (NT-proBNP) is a well-known biomarker for the diagnosis and prognosis of ADHF, which has been linked to the occurrence and prognosis of CRS-1 [11–13]. However, most studies focused on blood NT-proBNP and none of them discussed urine NT-proBNP (uNT-proBNP) as a predictor of CRS-1 in ADHF patients. Enkephalin is an endogenous opioid peptide that regulates a variety of pathophysiological processes. Nevertheless, enkephalin is unstable and difficult to measure in plasma. As a precursor and surrogate marker of enkephalin, proenkephalin (PENK) is stable and simple to measure in plasma [14]. In recent years, PENK has gradually come into people’s awareness as a new biomarker for predicting the kidney dysfunction and prognosis in patients with ADHF [15,16]. However, it is still unknown whether PENK can early predict the occurrence of CRS-1. Therefore, this study first analyzes the uNT-proBNP and plasma PENK (pPENK) levels in ADHF patients on admission, explores whether they can early predict the occurrence of CRS-1, and finally evaluates the vulnerable-phase prognostic value of uNT-proBNP, pPENK, and CRS-1 for ADHF patients.

## Methods

This study complied with the Declaration of Helsinki and was approved by the ethics committee of The First Hospital of Hebei Medical University (No.20201101). All subjects provided written informed consent.

### Study design and population

This is a prospective, double-center, observational study in a real-world setting. Consecutive hospitalized patients with ADHF [17] from The First Hospital of Hebei Medical University and The Second Hospital of Hebei Medical University were enrolled. ADHF was diagnosed based on the current guidelines criteria [18], showing typical symptoms and/or signs of ADHF and plasma B-type natriuretic peptide (pBNP) >100 pg/mL. AKI was defined according to the KDIGO criteria [5] as an increase in sCr of ≥ 26.5 mol/L (0.3 mg/dL) within 48 h or an increase in SCr by ≥ 50% within 7 d. For the homogeneity and reliability of the sCr tests that we can control and refer to the prior literature [6,19], the lowest value of sCr monitored during hospitalization and follow-up was defined as the baseline. We did not use oliguria for AKI diagnosis because its measurement is inaccurate in non-ICU or no-CCU wards. The inclusion criteria were as follows: (1) age ≥ 18 years; (2) enrollment within 1 h after admission for ADHF; (3) hospital stay time > 48 h. The exclusion criteria included: (1) on dialysis or needing emergency dialysis; (2) cardiogenic shock, acute myocarditis, acute aortic dissection, or concomitant terminal disease; (3) end-stage kidney disease, urinary tract infections or obstruction; (4) autoimmune diseases, sepsis, or surgery within one month; (5) heart or kidney transplantation; (6) exposure to nephrotoxic substances within 1 month (e.g., chemotherapy, radiotherapy, or contrast agents).

### Data collection

Based on whether or not AKI occurred during hospitalization, patients were divided into CRS-1 and no-CRS-1 groups. The following clinical data were collected on admission: demographics, medical history, laboratory data, chest X-rays indicators, and echocardiogram results. The medical treatment during hospitalization was also recorded.

### Sample collection and biomarker assays

On admission of each patient before any treatment, venous blood samples were collected from the patients into 5-mL ethylenediaminetetra-acetic acid (EDTA)-K2 anti-coagulation tubes and spot urine samples were collected into 10-mL standard urine collection tubes. Plasma and urine samples were separated after centrifugation at 5000 rpm and 4 °C for 10 min. All samples were then immediately stored in cryotubes at −80 °C until assayed. Before analysis, the urine samples were centrifuged twice at 134,000 rpm for 30 min at 4 °C to avoid potential interference caused by salt precipitation in urine. The pBNP concentrations were determined using an immunoassay (Alere Triage MeterPro, DE) at admission. The plasma NT-proBNP (pNT-proBNP) and uNT-proBNP levels were measured using Elecsys immunoassays on a Roche Cobas E411 (Roche Diagnostics; Grenzach, Germany) following the manufacturer’s instruction. The analytical range of NT-proBNP was 5–35,000 pg/mL and the coefficient of intra-assay variation was 3.0%. The pPENK was measured using commercially available human-specific enzyme-linked immunoassays (ELISA) kits (Elisa Biotech; Shanghai, China) on an automatic enzyme labeling instrument (VERSAmax, Sunnyvale, CA). Since the plasma neutrophil gelatinase-associated lipocalin (pNGAL) was proved as typical biomarker of early AKI and outcome in ADHF [20], it was used as the reference marker for comparisons with other biomarkers. The pNGAL was measured using the same method as pPENK. Other laboratory results were available in the hospital laboratory, and blood and urine creatinine were measured on admission and at least once per day during the first 7 d. To correct any potential variation in urine concentrations, the uNT-proBNP/urine creatinine (uCr) ratio (pg/nmol) was calculated in each identical sample [21,22]. All the patients underwent electrocardiogram, chest X-rays indicators, and echocardiogram examination on admission.

### Follow-up and endpoints

All included patients were followed from admission to 90 d after discharge (i.e., vulnerable phase) through medical chart review, telephone, or the instant messaging app WeChat. The primary endpoint was the occurrence of CRS-1 during hospitalization and the second endpoint was the composite of HF readmission or all-cause death 90 d after discharge.

### Sample size

Based on previous literature, we assume that the incidence of CRS-1 in ADHF patients is 25% [2] and the predictive validity of the area under the receiver operating characteristic curve (AUROC) for uNT-proBNP or pPENK is 0.70. The test efficiency is set as 1 − *β* = 0.8, the confidence level is *α* = 0.05, and the bilateral *Z* test level is 0.05. PASS version 15.0 software (NCSS, LLC., Kaysville, UT, USA) was used to calculate the sample size of the CRS-1 positive (*N*1 = 21) and negative (*N*2 = 63) groups. At least 84 patients were included in our study. Considering the complex clinical situation and the possibility of subjects falling off, we preset the falling-off rate to be about 15%. In this study, 100 hospitalized patients with ADHF should be included. We anticipated that the estimates would be reliable since our sample size and the number of outcome events exceeded the values calculated by the software.

### Statistical analysis

SPSS version 26.0 software (SPSS Inc., Chicago, IL) was used for all analyses. Categorical variables were expressed as counts (percentage) and compared by Pearson’s Chi-square test or Fisher’s exact test. Continuous variables with normal distribution were expressed as mean ± standard deviation (*SD*) or median and interquartile range, which were compared by Student’s *t*-test or nonparametric test (Mann–Whitney *U*-test) respectively. If necessary, continuous variables without normal distribution can be converted to a normal distribution by logarithmic transformation. Pearson’s correlation coefficients were calculated to determine the relationships between the pPENK or uNT-proBNP/uCr ratio and the pNGAL. The risk predictors for CRS-1 onset were determined by logistic regression analysis. The AUROC with 95% confidence intervals (CIs) were calculated to assess the early diagnostic value of pPENK and uNT-proBNP/uCr. The cutoff value was defined for the maximum Youden index. AUROCs were compared using the *Z* test with MedCalc version 20.0.3 software (MedCalc, Mariakerke, Belgium). The trend test was used to demonstrate the association between the incidence of CRS-1 and the pPENK or uNT-proBNP/uCr ratio. The Cox regression analysis was performed to evaluate the prognostic risk of pPENK, uNT-proBNP/uCr ratio, and CRS-1 for the 90-d clinical outcome and the cumulative hazard curves were plotted according to the grouping of each variable based on the low and high levels. A 2-sided *p* value of < 0.05 was considered statistically significant.

## Results

### Patient baseline characteristics

**Figure 1.**
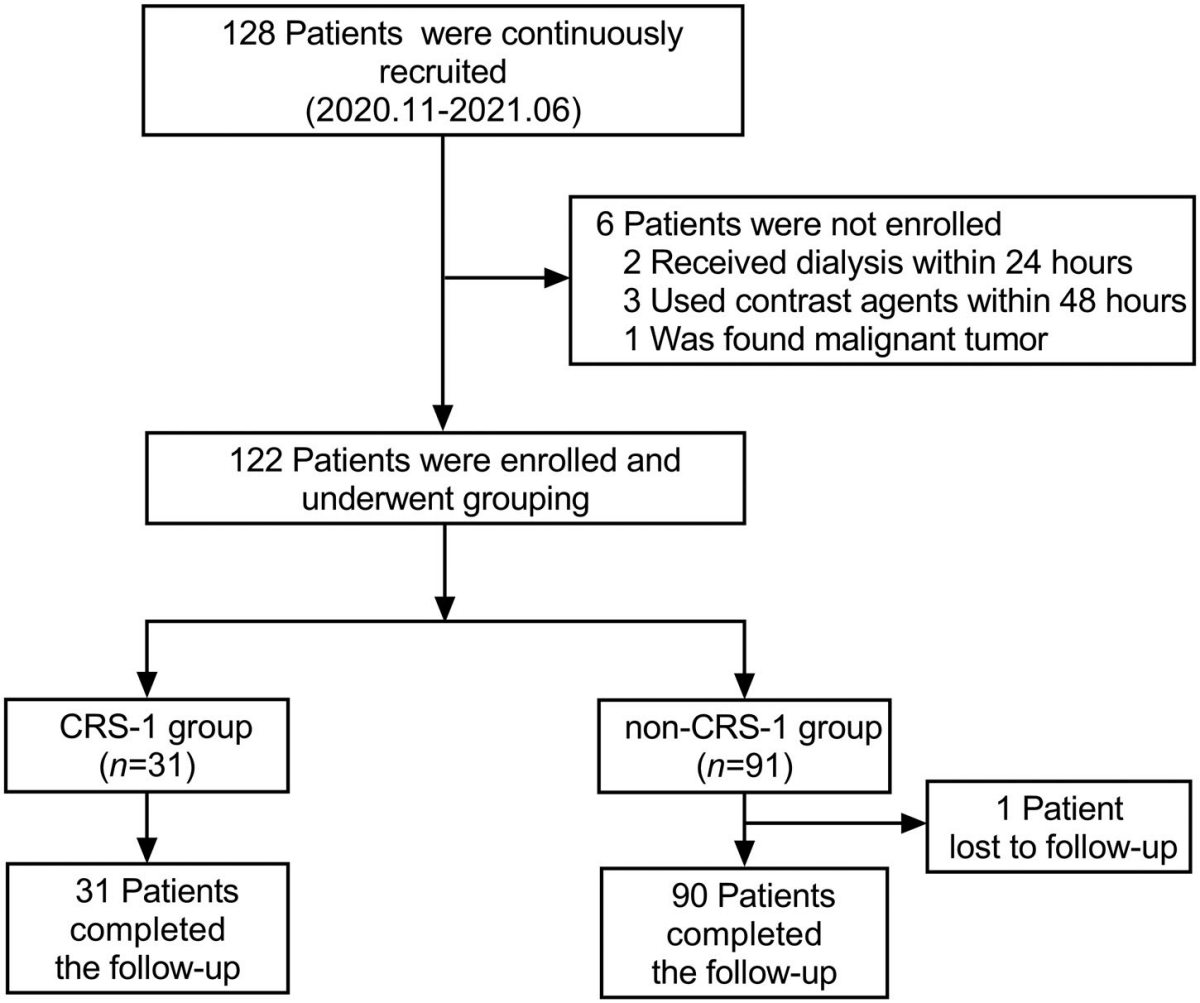
Recruiting, grouping, and follow-up of study population. CRS-1: cardiorenal syndrome type 1.

A total of 128 hospitalized ADHF patients were prospectively and consecutively recruited from November 2020 to June 2021. Among them, two patients received dialysis within 24 h of admission, three patients underwent coronary angiography with contrast agents within 48 h, one patient had a malignant tumor discovered during hospitalization, and one patient was lost to follow-up. Finally, 121 subjects completed the study and follow-up (Figure 1). Patients were divided into the CRS-1 group and the non-CRS-1 group depending on whether or not CRS-1 has developed. The baseline characteristic data of patients are shown in Table 1.

**Table 1.** Baseline characteristics and outcomes of the CRS-1 and no-CRS-1 cohort in ADHF patients.

Characteristic	CRS-1 (*n* = 31)	No-CRS-1 (*n* = 90)	*t*/*Z*/*χ*^2^	*p*
Age, years	68.3 ± 13.2	65.8 ± 12.5	−0.757	0.450
Female, *n* (%)	9 (29.0)	40 (44.4)	2.273	0.132
Body mass index, kg/m^2^	24.1 ± 4.1	24.9 ± 3.6	−1.029	0.305
Smoking, *n* (%)	17 (54.8)	31 (34.4)	4.007	0.045
Recurrent heart failure, *n* (%)	19 (61.3)	36 (40.0)	4.215	0.040
Systolic blood pressure, mmHg	134.9 ± 35.8	131.3 ± 26.7	0.513	0.611
Etiology, *n* (%)				
Ischemic	13 (41.9)	40 (44.4)	0.059	0.805
Hypertensive	1 (3.2)	3 (3.3)	0.001	0.977
Cardiomyopathy	9 (29.0)	17 (18.9)	1.406	0.236
NYHA class IV, *n* (%)	17 (54.8)	26 (28.9)	6.778	0.009
Medical history, *n* (%)				
Hypertension	23 (74.2)	55 (61.1)	1.723	0.189
Coronary heart disease	16 (51.6)	31 (34.4)	2.861	0.091
Diabetes mellitus	13 (41.9)	19 (21.1)	5.140	0.023
Chronic kidney disease	12 (38.7)	4 (4.4)	<0.001	<0.001
Stage 2	1 (3.2)	2 (2.2)	–	–
Stage 3	2 (6.4)	2 (2.2)	–	–
Stage 4	9 (29.0)	0 (0.0)	–	–
Pulmonary rales *n* (%)	18 (58.1)	31 (34.4)	5.339	0.021
Laboratory findings				
Hemoglobin , g/L (Ref: 130 ∼ 175)	116.5 ± 26.0	134.2 ± 24.8	−3.385	0.001
uCr, mmol/L (Ref: 5300.0 ∼ 18000.0)	4741.0 (2846.0, 6625.0)	7775.0 (4512.0, 11222.3)	−2.737	0.006
Blood creatinine, μmol/L (Ref: 57.0 ∼ 111.0)	172.7 ± 104.1	79.3 ± 23.8	4.951	<0.001
Blood urea nitrogen, mmol/L (Ref: 3.60 ∼ 9.50)	14.01 ± 9.11	6.69 ± 2.71	4.407	<0.001
eGFR, mL/min/1.73 m^2^ (Ref: 80.0 ∼ 120.0)	55.2 (30.6, 72.3)	66.1 (44.7, 79.5)	–3.937	<0.001
Sodium, mmol/L (Ref: 137.0 ∼ 147.0)	136.2 ± 5.2	140.8 ± 4.0	–7.709	<0.001
Total cholesterol, mmol/L (Ref: 2.80 ∼ 5.17)	3.65 ± 1.14	4.13 ± 1.14	–2.022	0.045
LDL-C, mmol/L (Ref: <3.37)	2.30 ± 0.82	2.59 ± 0.92	–1.554	0.123
HbA1c, % (Ref: 3.0 ∼ 6.0)	7.14 ± 1.44	6.74 ± 1.74	1.515	0.252
cTnI, ng/mL (Ref: <0.02)	0.08 (0.04, 0.58)	0.09 (0.04, 1.77)	–0.375	0.707
Plasma BNP, pg/m (Ref: <100.0)	1818.0 (1124.5, 3230.0)	678.5 (305.3, 1137.9)	–4.562	<0.001
Plasma NT-proBNP, pg/mL (Ref: <300.0)	8106.0 (4220.0, 17800.0)	2099.4 (1003.6, 5136.8)	–5.267	<0.001
Urinary NT-proBNP, pg/mL	2375.0 (922.8, 4416.0)	151.5 (57.0, 314.7)	–7.184	<0.001
uNT-proBNP/uCr ratio, pg/nmol	415.8 (224.7, 1036.1)	22.6 (7.8, 54.3)	–7.184	<0.001
Plasma NGAL, ng/mL	93.0 ± 69.0	35.8 ± 16.8	6.966	<0.001
Plasma PENK, pg/mL	90.8 (60.6, 145.5)	38.3 (27.1, 52.6)	–6.612	<0.001
Pulmonary edema (X-ray), *n* (%)	19 (61.3)	34 (37.8)	5.179	0.023
Echocardiogram				
LVEF, %	40.9 ± 13.0	48.9 ± 15.4	–2.590	0.011
Left atrial diameter, mm	47.0 (41.0, 50.0)	41.0 (37.0, 47.3)	–2.869	0.004
LVEDD, mm	62.5 ± 12.1	53.6 ± 11.2	3.738	<0.001
Treatment, *n* (%)				
Loop diuretics	30 (96.8)	89 (98.9)	0.448	0.448
ARA	30 (96.8)	86 (95.6)	1.000	0.620
Beta-blocker	21 (67.7)	64 (71.1)	0.125	0.723
ACEI or ARB	11 (35.5)	29 (32.2)	0.111	0.739
ARNI	14 (45.2)	23 (25.6)	4.175	0.041
rh-BNP	21 (67.7)	45(50.0)	2.927	0.087
SGLT2i	2 (6.5)	11 (12.2)	0.511	0.300
Outcomes 90 d after discharge, *n* (%)				
All-cause Death	6 (19.4)	1 (1.1)	0.001	0.001
HF readmission	6 (19.4)	9 (10.0)	0.208	0.148
All-cause death or HF readmission	12 (38.7)	10 (11.1)	11.806	0.001

CRS-1: cardiorenal syndrome type 1; ADHF: acute decompensated heart failure; NYHA: New York Heart Association; Ref: reference range; uCr: urine creatinine; LDL-C: low density lipoprotein cholesterol; HbA1c: hemoglobin A1c; cTnI: cardiac troponin I; BNP: brain natriuretic peptide; NT-proBNP: N-terminal pro-brain natriuretic peptide; uNT-proBNP: urine N-terminal pro-brain natriuretic peptide; NGAL: neutrophil gelatinase-associated lipocalin; PENK: proenkephalin; LVEF: left ventricular ejection fraction; LVEDD: left ventricular end-diastolic diameter; ACEI: angiotensin converting enzyme inhibitor; ARB: angiotensin receptor blocker; ARNI: sacubitril/valsartan; ARA: aldosterone receptor antagonists; rh-BNP: recombinant human brain natriuretic peptide; SGLT2i: Sodium–glucose cotransporter 2 inhibitors.

The age of the enrolled 121 patients was 66.5 ± 12.7 years; 49 (40.5%) patients were female; 48 (39.7%) were smoking; 55 (45.5%) were previously hospitalized for ADHF; 43 (35.5%) were NYHA class IV. The most common etiology was myocardial ischemia (53 patients; 43.8%) and the most common co-morbidity was hypertension (78 patients; 64.5%). Sixteen patients suffered from CKD (three patients in Stage 2, four patients in Stage 3, and nine patients in Stage 4). Ultimately, the incidence of CRS-1 in ADHF was 25.6% (31/121), with 19 patients in Stage 1 of AKI, 1 in Stage 2, 2 in Stage 3, and 9 in Stage 4. Moreover, 9.9% (12/121) of patients developed acute-on-chronic AKI and 15.79% (3/19) of AKI patients developed into CKD. The clinical outcome of the composite of HF readmission or all-cause death 90 d after discharge were 38.7% (12/31) and 11.1% (10/90) in the CRS-1 group and no-CRS-1 group, respectively (*p* = 0.001).

### Levels of pNGAL, pBNP, pNT-proBNP, uNT-proBNP/uCr ratio, and pPENK

**Figure 2.**
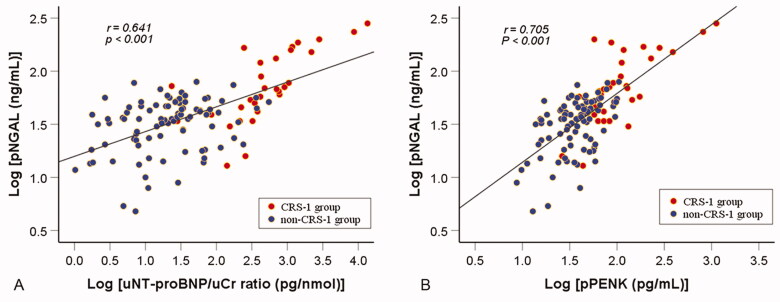
Association between the pNGAL and the uNT-proBNP/uCr ratio or pPENK in ADHF patients. After undergoing logarithmic transformation, there was a positive linear correlation between pNGAL and nT-probNP /uCr ratio (A) or pPENK (B). pNGAL: plasma neutrophil gelatinase-associated lipocalin; uNT-proBNP: urine N-terminal pro-B-type natriuretic peptide; uCr: urine creatinine; pPENK: plasma proenkephalin; CRS-1: cardiorenal syndrome type 1.

As the reference biomarker, the level of pNGAL was significantly higher in the CRS-1 group than that of in the non-CRS-1 group (*p* < 0.001). Furthermore, the CRS-1 group had significantly elevated levels of pBNP, pNT-proBNP, uNT-proBNP/uCr ratio, and pPENK (*p* < 0.001). In addition, after logarithmic transformation (to convert the data to a normal distribution), the level of the pNGAL was positively correlated with both uNT-proBNP/uCr ratio (*r* = 0.641, *p* < 0.001) (Figure 2(A)) and pPENK (*r* = 0.705, *p* < 0.001) (Figure 2(B)).

### Predictive value of pPENK and uNT-proBNP/uCr ratio for the occurrence of CRS-1

The logistic regression was used to uncover the predictive value of the uNT-proBNP/uCr ratio and pPENK for the occurrence of CRS-1 in ADHF patients (Table 2). In the univariate analysis, high-level of pBNP, pNT-proBNP, uNT-proBNP/uCr, pNGAL, and pPENK were all risk predictor for CRS-1 onset, while in the multivariate analysis only the high-level of uNT-proBNP/uCr ratio [OR 1.015 (95% CI 1.003–1.028), *p* = 0.012] and pPENK [OR 1.093 (95% CI 1.022–1.169), *p* = 0.010] were independently associated with subsequent CRS-1.

**Table 2.** Univariate and multivariate logistic regression analysis of CRS-1 occurrence in ADHF patients.

Variables	Univariate			Multivariate		
	OR	95% CI	*p* Value	OR	95% CI	*p* Value
Age > 65 years	1.157	0.502–2.667	0.732	–	–	–
Sex (female)	1.956	0.811–4.714	0.135	–	–	–
Body mass index <20 kg/m^2^	1.460	0.643–3.312	0.366	–	–	–
Systolic blood pressure ≥ 140 mmHg	1.647	0.717–3.785	0.240	–	–	–
Atrial fibrillation	1.824	0.790–4.209	0.159	–	–	–
Smoking	2.311	1.007–5.302	0.048	3.717	0.947–14.589	0.060
NYHA class IV	2.989	1.288–6.934	0.011	0.624	0.163–2.391	0.492
Diabetes mellitus	2.699	1.125–6.473	0.026	2.446	0.536–11.164	0.248
Chronic kidney disease	13.579	3.946–46.729	<0.001	4.903	0.927–25.942	0.061
Serum creatinine > 90 μmol/L	9.332	2.647–32.915	<0.001	4.559	0.792–26.241	0.089
Plasma BNP > 1000 pg/m	8.333	3.087–22.493	<0.001	3.284	0.572–18.848	0.182
Plasma NT-proBNP > 5000 pg/mL	6.117	2.513–14.891	<0.001	1.036	0.572–18.848	0.969
uNT-proBNP/uCr > 30 pg/nmol	12.772	3.616–45.117	<0.001	5.153	1.012–26.232	0.048
Plasma NGAL > 40 ng/mL	5.143	2.006–13.187	<0.001	3.319	0.769–14.322	0.108
Plasma PENK > 45 pg/mL	10.125	3.266–31.393	<0.001	8.503	1.683–42.967	0.010

CRS-1: cardiorenal syndrome type 1; ADHF: acute decompensated heart failure; Ref: reference range; BNP: brain natriuretic peptide; NT-proBNP: N-terminal pro-brain natriuretic peptide; uNT-proBNP: Urine N-terminal pro-brain natriuretic peptide; uCr: urine creatinine; NGAL: neutrophil gelatinase-associated lipocalin; PENK: proenkephalin.

The cutoff values of age, body mass index, systolic blood pressure, serum creatinine, plasma BNP, plasma NT-proBNP, uNT-proBNP/uCr, plasma NGAL, and plasma PENK were calculated with the Youden index based on the predictive power of each variable for the CRS-1 occurrence.

**Figure 3.**
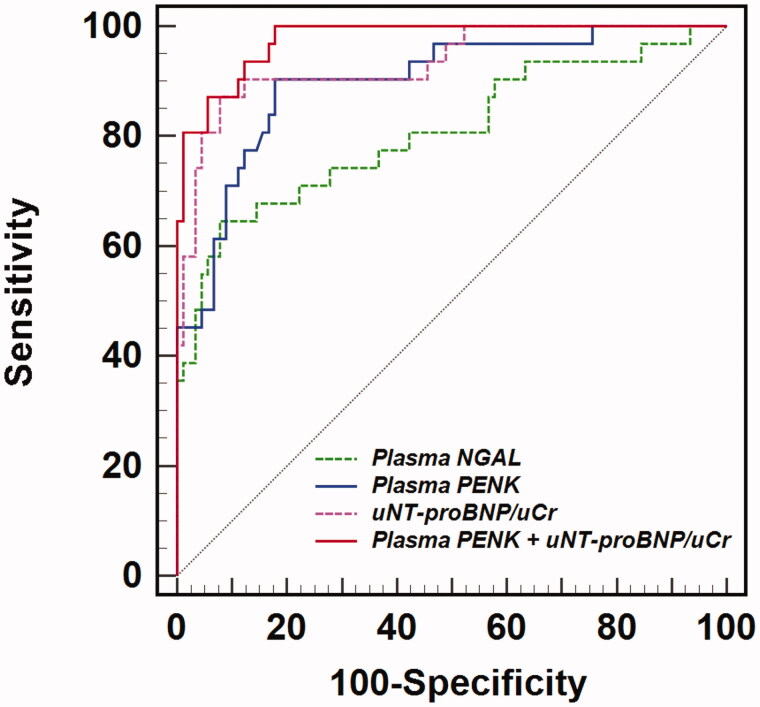
The receiver operating curves (ROC) of plasma NGAL, plasma PENK, uNT-proBNP/uCr ratio, and plasma PENK combined with uNT-proBNP/uCr ratio for predicting CRS-1 in ADHF patients. NGAL: neutrophil gelatinase-associated lipocalin; PENK: plasma proenkephalin; uNT-proBNP: urine N-terminal pro-B-type natriuretic peptide; uCr: urine creatinine.

The optimal uTproBNP/uCr cutoff value for predicting CRS-1 was determined to be 119.0 pg/nmol (Youden index, 0.793). The uNT-proBNP/uCr presented a better diagnostic accuracy for CRS-1 onset than pPENK or pNGAL, with the higher AUROC of 0.934 (95% CI 0.874–0.971) (Figure 3, Tables 3 and 4). The AUROC of pPENK combined with uNT-proBNP/uCr was 0.976 (95% CI 0.930–0.995), with a sensitivity of 0.9677 and a specificity of 0.8222, which was significantly higher than the predictive value of pPENK or pNGAL alone (*p* < 0.05). However, it is not superior to the predictive value of uNT-proBNP/uCr alone (*p* > 0.05) (Table 4). The comparison of AUROCs for pNGAL, pENK, uNT-proBNP/uCr, and pPENK combined with uNT-proBNP/uCr was presented in Table 4.

**Table 3.** The area under the receiver operating curves (AUROC) of plasma NGAL, plasma PENK, plasma NT-proBNP, uNT-proBNP/uCr ratio, and plasma PENK + uNT-proBNP/uCr for predicting CRS-1 in ADHF patients.

Variables	Cutoff value	Youden index	AUROC (95% CI)	*P* Value	*Z*-statistic	Sensitivity	Specificity
Plasma NGAL	56.5	0.567	0.808 (0.726–0.874)	<0.001	5.900	0.6452	0.9222
Plasma PENK	57.0	0.725	0.899 (0.831–0.946)	<0.001	11.900	0.9032	0.8222
uNT-proBNP/uCr	119.0	0.793	0.934 (0.874–0.971)	<0.001	15.618	0.8710	0.9222
Plasma PENK + uNT-proBNP/uCr	–	0.822	0.976 (0.930–0.995)	<0.001	42.921	0.9677	0.8222

CRS-1: cardiorenal syndrome type 1; ADHF: acute decompensated heart failure; AUROC: area under the receiver operating curves; NT-proBNP: Amino-terminal pro-B-type natriuretic peptide; uNT-proBNP: urine NT-proBNP; uCr: urine creatinine; NGAL: neutrophil gelatinase-associated lipocalin; PENK: preproenkephalin.

**Table 4.** The comparison of area under the receiver operating curves (AUROC) between plasma NGAL, plasma PENK, uNT-proBNP/uCr ratio, and plasma PENK + uNT-proBNP/uCr for predicting CRS-1 in ADHF patients.

Variables	Comparison	Difference between AUROCs (95% CI)	Standard error	*Z*-statistic	*p* Value
Plasma PENK	*vs.* Plasma NGAL	0.092 (0.007–0.176)	0.043	0.043	0.033
UNT-proBNP/uCr	*vs.* Plasma NGAL	0.126 (0.019–0.233)	0.055	2.309	0.021
	*vs.* Plasma PENK	0.035 (−0.054–0.123)	0.045	0.764	0.445
Plasma PENK + uNT-proBNP/uCr	*vs.* Plasma NGAL	0.168 (0.079–0.257)	0.045	3.709	<0.001
	*vs.* Plasma PENK	0.076 (0.021–0.133)	0.029	2.701	0.007
	*vs.* uNT-proBNP/uCr	0.042 (−0.009–0.094)	0.026	1.602	0.109

CRS-1: cardiorenal syndrome type 1; ADHF: acute decompensated heart failure; AUROC: area under the receiver operating curves; NT-proBNP: Amino-terminal pro-B-type natriuretic peptide; uNT-proBNP: urine NT-proBNP; uCr: urine creatinine; NGAL: neutrophil gelatinase-associated lipocalin; PENK: preproenkephalin.

**Figure 4.**
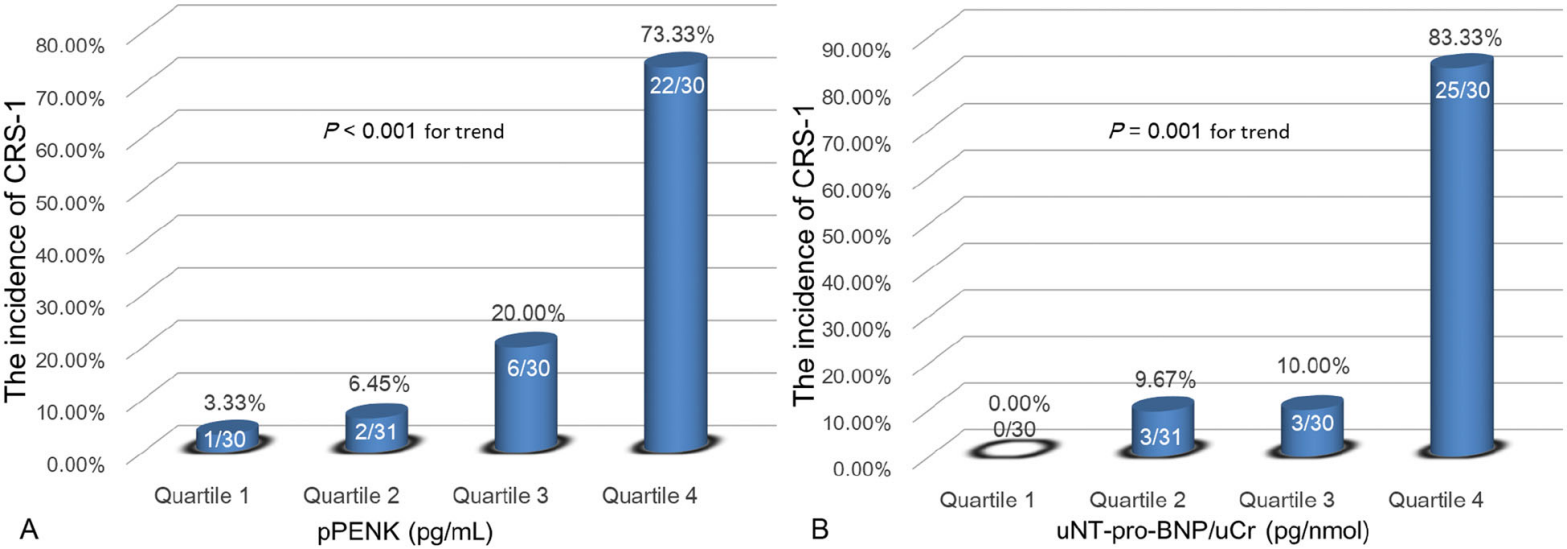
Association between incidence of CRS-1 and quartiles of the pPENK (A) and uNT-proBNP/uCr ratio (B) in ADHF patients. pPENK: plasma proenkephalin; uNT-proBNP: urine N-terminal pro-B-type natriuretic peptide; uCr: urine creatinine; CRS-1: cardiorenal syndrome type 1.

According to the quartiles of the pPENK (pg/mL) and uNT-proBNP/uCr ratio (pg/nmol), we further divided the study cohort into four patient groups respectively: (1) pPENK: Quartile 1, <29.58; Quartile 2, 29.58–46.17; Quartile 3, 46.18–72.50; and Quartile 4, >72.50. We found that the incidence of CRS-1 significantly increased with rising pPENK quartiles (3.33%, 6.45%, 20.00%, and 73.33%, respectively; *p* < 0.001 for trend) (Figure 4(A)). (2) uNT-proBNP/uCr: Quartile 1, <11.22; Quartile 2, 11.22–32.09; Quartile 3, 32.10–189.63; and Quartile 4, >189.63. The results indicate that the incidence of CRS-1 also significantly increased with increasing uNT-proBNP/uCr quartiles (0.00%, 9.67%, 10.00%, and 83.33%, respectively; *p* = 0.001 for trend) (Figure 4(B)).

### Prognostic value of pPENK, uNT-proBNP/uCR ratio, and CRS-1 for the clinic outcome of ADHF patients

We divided the study population into the positive and negative group based on whether patients had the composite outcome of HF readmission or all-cause death 90 d after discharge. According to the development of clinic outcomes the patients’ baseline characteristics are shown in Table 5.

**Table 5.** Baseline characteristics and CRS-1 of the outcome + and outcome – cohort in ADHF patients 90 d after discharge.

Characteristic	Outcome + (*n* = 22)	Outcome − (*n* = 99)	*t*/*Z*/*χ*^2^	*p*
Age, years	71.6 ± 9.6	65.3 ± 13.0	2.144	0.034
Female, *n* (%)	15 (68.2)	57 (57.6)	0.840	0.359
Body mass index, kg/m^2^	23.1 ± 3.5	25.1 ± 3.7	−2.315	0.022
Smoking, *n* (%)	9 (40.9)	39 (39.4)	0.017	0.895
Recurrent heart failure, *n* (%)	14 (63.6)	41 (41.4)	3.585	0.058
Systolic blood pressure, mmHg	121.8 ± 22.4	134.6 ± 29.8	−1.897	0.060
Etiology, *n* (%)				
Ischemic	7 (31.8)	47 (47.5)	1.786	0.181
Hypertensive	2 (9.1)	2 (2.0)	0.151	0.151
Cardiomyopathy	7 (31.8)	19 (19.2)	0.250	0.154
NYHA class IV, *n* (%)	12 (54.5)	31 (31.3)	4.241	0.039
Medical history, *n* (%)				
Hypertension	12 (54.5)	66 (66.7)	1.154	0.283
Coronary heart disease	7 (31.8)	40 (40.4)	0.559	0.455
Diabetes mellitus	5 (22.7)	27 (27.3)	0.191	0.662
Chronic kidney disease	7 (31.8)	9 (9.1)	0.010	0.010
Stage 2	2 (9.1)	6 (6.0)	–	–
Stage 3	3 (13.6)	2 (2.0))	–	–
Stage 4	2 (9.1)	1 (1.0)	–	–
Pulmonary rales *n* (%)	16 (72.7)	33 (33.3)	11.592	0.001
Laboratory findings				
Hemoglobin , g/L (Ref: 130 ∼ 175)	115.6 ± 25.4	132.8 ± 25.4	−2.873	0.005
uCr, mmol/L (Ref: 5300.0 ∼ 18,000.0)	5976.5 (3121.3, 10905.0)	6339.0 (4136.0, 10131.0)	−0.222	0.824
Blood creatinine, μmol/L (Ref: 57.0 ∼ 111.0)	150.3 ± 101.9	92.8 ± 55.2	2.564	0.017
Blood urea nitrogen, mmol/L (Ref: 3.60 ∼ 9.50)	12.6 ± 8.8	7.7 ± 4.9	2.526	0.019
eGFR, mL/min/1.73 m^2^ (Ref: 80.0 ∼ 120.0)	62.4 (36.3, 70.8)	65.8 (43.6, 75.7)	−2.352	0.021
Sodium, mmol/L (Ref: 137.0 ∼ 147.0)	139.3 ± 5.1	140.6 ± 4.1	−1.285	0.201
Total cholesterol, mmol/L(Ref: 2.80 ∼ 5.17)	3.61 ± 1.15	4.10 ± 1.14	−1.861	0.065
LDL-C, mmol/L (Ref: <3.37)	2.30 ± 0.90	2.56 ± 0.90	−1.226	0.223
HbA1c, % (Ref: 3.0 ∼ 6.0)	6.86 ± 1.94	6.84 ± 1.62	0.050	0.960
cTnI, ng/mL (Ref: <0.02)	0.21 (0.03, 2.014)	0.07 (0.04, 1.29)	−0.509	0.611
Plasma BNP, pg/m (Ref: <100.0)	1815.0 (1042.1, 3050.0)	805.0 (332.0, 1340.0)	−3.253	0.001
Plasma NT-proBNP, pg/mL (Ref: <300.0)	7987.0 (3500.6, 17,738.5)	2409.0 (1143.0, 5599.7)	−3.390	0.001
Urine NT-proBNP, pg/mL	1156.6 (485.3, 3988.5)	187.5 (66.3, 387.6)	−4.005	<0.001
uNT-proBNP/uCr ratio, pg/nmol	229.8 (32.2, 506.2)	29.2 (9.9, 80.90)	−3.179	0.0001
Plasma NGAL, ng/mL	49.7 (30.6, 75.1)	38.7 (24.7, 52.1)	−1.492	0.136
Plasma PENK, pg/mL	57.3 (39.5, 85.2)	44.6 (28.3, 67.7)	−1.441	0.149
Pulmonary congestion (X-ray), *n* (%)	11 (50.0)	34 (34.3)	1.889	0.169
Echocardiogram				
LVEF, %	42.7 ± 16.2	47.8 ± 14.9	−1.429	0.156
Left atrial diameter, mm	48.1 ± 11.9	45.5 ± 30.3	0.656	0.514
LVEDD, mm	61.4 ± 16.6	54.6 ± 10.4	3.405	0.001
Treatment, *n* (%)				
Loop diuretics	21 (95.5)	98 (99.0)	0.332	0.332
ARA	21 (95.5)	95 (96.0)	1.000	0.640
Beta-blocker	12 (54.5)	73 (73.7)	3.172	0.075
ACEI or ARB	5 (22.7)	35 (35.4)	1.297	0.255
ARNI	9 (40.9)	28 (28.3)	1.352	0.245
rh-BNP	16 (72.7)	50 (50.5)	3.585	0.058
SGLT2i	0 (0.0)	13 (13.1)	0.123	0.063
CRS-1, *n* (%)	12 (54.4)	19 (19.2)	11.806	0.001

CRS-1: cardiorenal syndrome type 1; ADHF: acute decompensated heart failure; NYHA: New York Heart Association; Ref: reference range; uCr: urine creatinine; LDL-C: low density lipoprotein cholesterol; HbA1c: hemoglobin A1c; cTnI: cardiac troponin I; BNP: brain natriuretic peptide; NT-proBNP: N-terminal pro-brain natriuretic peptide; uNT-proBNP: urine NT-proBNP; NGAL: neutrophil gelatinase- associated lipocalin; PENK: preproenkephalin; LVEF: left ventricular ejection fraction; LVEDD: left ventricular end-diastolic diameter; ACEI: angiotensin converting enzyme inhibitor; ARB: angiotensin receptor blocker; ARNI: sacubitril/valsartan; ARA: aldosterone receptor antagonists; rh-BNP: recombinant human brain natriuretic peptide; SGLT2i: Sodium–glucose cotransporter 2 inhibitors; CRS-1: cardiorenal syndrome type 1; Outcome +: patients with the composite of HF readmission or all-cause death 90 d after discharge; Outcome −: patients without the composite of HF readmission or all-cause death 90 d after discharge.

Besides, the multivariate Cox regression analysis suggested that the pPENK [HR 1.014 (95% CI 1.000–1.042), *p* = 0.044] and uNT-proBNP/uCr ratio [HR 0.998 (95% CI 0.997–1.000), *p* = 0.045] were independent predictors for adverse outcome in ADHF patients 90 d after discharge (Table 6).

**Table 6.** Multivariate cox regression analysis for positive outcome in ADHF patients 90 d after discharge.

Variables	HR	95% CI	*p* Value
Body mass index, kg/m^2^	1.235	0.965–1.582	0.094
Chronic kidney disease history	1.710	0.452–6.460	0.429
Anemia	1.683	0.411–6.892	0.470
Plasma BNP, pg/m	1.001	1.000–1.002	0.008
Plasma NT-proBNP, pg/mL	1.000	1.000–1.000	0.942
uNT-proBNP/uCr, pg/nmol	0.998	0.997–1.000	0.045
Plasma NGAL, ng/mL	1.011	0.987–1.035	0.379
Plasma PENK, pg/mL	1.014	1.000–1.028	0.044
LVEDD, mm	1.235	0.965–1.582	0.096
CRS-1	1.669	0.297–9.374	0.561

CRS-1: cardiorenal syndrome type 1; ADHF: acute decompensated heart failure; BNP: brain natriuretic peptide; NT-proBNP: N-terminal pro-brain natriuretic peptide; uNT-proBNP: urine N-terminal pro-brain natriuretic peptide; uCr: urine creatinine; PENK: proenkephalin; NGAL: neutrophil gelatinase-associated lipocalin; LVEDD: left ventricular end-diastolic diameter.

**Figure 5.**
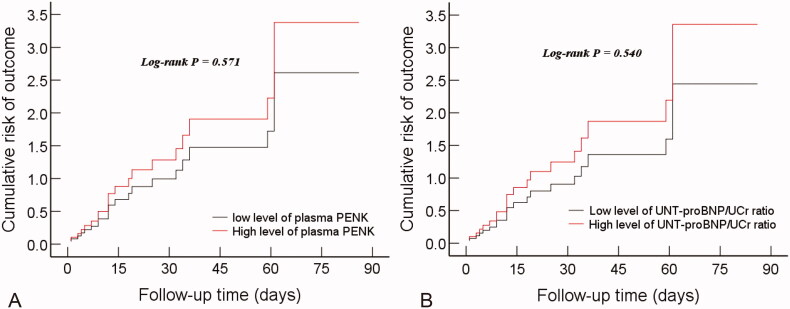
The cumulative risk curve of the pPENK (A) and uNT-proBNP/uCr ratio (B) for the composite-outcome of all-cause death or HF readmission 90 d after discharge in ADHF patients. uNT-proBNP: urine N-terminal pro-B-type natriuretic peptide; uCr: urine creatinine; pPENK: plasma proenkephalin.

We further divided all patients into high-level and low-level groups by the cutoff values of the pPENK and uNT-proBNP/uCr ratio respectively. Although the cumulative risk curve showed that both high-level pPENK (Figure 5(A)) and uNT-proBNP/uCr ratio (Figure 5(B)) increased the risk of the composite-outcome of HF readmission or all-cause death 90 d after discharge, the difference between the high-level group and the low-level group was not statistically significant (log-rank *p* > 0.05).

## Discussion

To our knowledge, this is the first study to examine the predictive ability of pPENK and uNT-proBNP for CSR-1. The major findings of this study were as follows: (1) the pPENK and uNT-proBNP/uCr ratio but not pBNP, pNT-proBNP, or pNGAL levels were strong independent predictors for CRS-1 in hospitalized patients with ADHF; (2) the incidence of CRS-1 significantly increased with increasing pPENK or uNT-proBNP/uCr ratio; (3) there was a strong positive linear correlation between the pPENK concentration or uNT-proBNP/uCr ratio and pNGAL; (4) the pPENK and uNT-proBNP/uCr levels were significantly different between the composite-outcome negative and positive groups; (5) the pPENK and uNT-proBNP/uCr were independent predictors of the risk of HF readmission or all-cause death 90 d after discharge in ADHF patients.

The pre-proBNP precursor with 134 amino acids was converted to the proBNP with 108 amino acids by removing a signal peptide with 26 amino acids. Then proBNP was further split into two molecular forms: biologically active BNP and inactive NT-proBNP [23]. NT-proBNP is mainly eliminated by the kidney, thus, it could be measured in both plasma and urine in HF patients [18,22,23]. Previous studies have confirmed that pNT-proBNP was a predictor of CRS-1 [17,18,20]. Yet few studies have investigated the efficacy of uNT-proBNP as a biomarker for predicting the occurrence of CRS-1 in ADHF patients. Our data indicated that the level of uNT-proBNP/uCr in the CRS-1 group was significantly higher than that in the non-CRS-1 group (*p* < 0.05). Moreover, a strong positive linear correlation was observed between the uNT-proBNP/uCr and the pNGAL. These findings suggested that uNT-proBNP can be used as an alternative for pNGAL to evaluate the occurrence of CRS-1 and the prognosis in ADHF patients.

The PENK with 41 amino acids (PENK A 119–159) is one of the proteolytic products of the pre-PENK with 267 amino acids, which exhibits various functions, including the involvement in stress response mechanisms, antinociception, and immune stimulation [14]. Furthermore, PENK is a stable endogenous opioid peptide, a surrogate for enkephalins and is produced throughout the human body, including the heart and kidney [14,24,25]. Previous studies on PENK showed that the level of PENK was higher in the HF population than that in the healthy population and was closely related to the deterioration of kidney function, the severity of HF, and mortality. However, only limited prognostic information was provided for patients with acute HF [15,25]. On the other hand, some studies suggested that the PENK level can reflect the heart and kidney status in acute HF patients, but the correlation was no longer independent after adjustment for other renal markers (including sCr) [16]. Our statistical results were in agreement with the finding that baseline pPENK was strongly correlated with baseline sCr [16]. Our data also demonstrated a linear correlation between pPENK and pNGAL. Moreover, pPENK could independently predict the occurrence of CRS-1 and the composite of HF readmission or all-cause death 90 d after discharge in ADHF, which was partially inconsistent with previous literature [16]. The reasons are unknown and need to be further explored.

Currently, the general standard for diagnosing CRS-1 is still an acute increase in sCr [5]. However, the level of sCr in AKI does not rise up significantly until 72 h after the initial insult to the kidney [20,26]. More extensive studies on new biomarkers are needed for predicting the occurrence of CRS-1 as early as possible. Studies tried to use NGAL to differentiate between various types of stable CKD, but failed [27], whereas pNGAL was proved to be able to predict the AKI occurrence and outcome in ADHF, but the reliability of its predictive value remains controversial [6,28], and more investigations are still needed before its wide application in clinical practice. It is, therefore, necessary to explore more novel biomarkers. We evaluated the predictive value of pPENK and uNT-proBNP on the occurrence of CRS-1 in ADHF patients and compared them with the reference pNGAL. Our results showed that pPENK and uNT-proBNP/uCr on admission were two independent predictors of CRS-1 in hospitalized ADHF patients. The higher AUROCs of pPENK and uNT-proBNP/uCr demonstrated a higher accuracy in early predicting CRS-1 in ADHF patients. There was no significant difference in AUROCs between the pPENK and uNT-proBNP/uCr. In other words, the pPENK and uNT-proBNP/uCr show almost the same ability to ideally early predict CRS-1. By contrast, the pNGAL level was not an independent predictor of CRS-1 in ADHF, and as the reference biomarker, it presented slightly lower predictive power due to the lower AUROC (Figure 3 and Table 3). Furthermore, the incidence of CRS-1 significantly increased as the pPENK or uNT-proBNP/uCr rose (Figure 4). Our findings provided powerful evidence for pPENK and uNT-proBNP to predict the CRS-1 onset in ADHF patients.

Higher levels of pPENK [15,25] and uNT-proBNP [22,29,30] have been investigated as predictors of poor prognosis in HF patients. Our Cox regression analysis suggested that the pPENK and uNT-proBNP/uCr ratio were two independent predictors of adverse outcomes in ADHF patients 90 d after discharge (vulnerable-phase). Although the statistical difference was not obvious, the cumulative risk curves revealed that both high-level pPENK and uNT-proBNP/uCr ratio increased the risk of the composite outcome of HF readmission or all-cause death 90 d after discharge, which may require longer follow-up time for further observation in the future. In addition, studies have demonstrated that AKI was associated with a worse outcome for patients with ADHF [31,32]. Our data further indicated an independent correlation between the CRS-1occurrence and poor prognosis in the HF vulnerable phase. Moreover, the cumulative risk curves showed that the CRS-1 group significantly increased the risk of the composite outcomes in comparison to the non-CRS-1 group (Figure 5(C)).

It is also noteworthy that few biomarker studies have thoroughly investigated the prediction of CRS-1 in ADHF patients with chronic kidney disease (CKD). Our findings support that CKD was an independent predictor of CRS-1 in ADHF populations. Other studies reported that patients with acute CRS were at greater risk of mortality and morbidity when they had a history of CKD [33]. Our Cox regression analysis, however, did not support that CKD is an independent risk predictor of poor vulnerable-phase prognosis in ADHF patients. Although some controversies still exist on the prognostic value of CKD in ADHF patients, CKD must not be ignored in ADHF patients. Early prediction of CRS-1 in these patients remains a challenge. Furthermore, it should be noted that nine CKD patients in stage 4 developed AKI in our study, and the use of diuretics and ACEI/ARB/ARNI was limited in these patients. However, these patients did not eventually progress to renal replacement therapy. The reason may be that most patients used rhBNP, which could be diuretic and vasodilator, resist renin–angiotensin–aldosterone system (RAAS), and improve the eGFR, thereby improving cardiac and kidney function in patients with ADHF [18,34].

Another issue to keep in mind is that patients who had CRS-1 had higher blood creatinine levels on admission, suggesting that baseline creatinine was probably also a predictor of CRS-1. In fact, we once analyzed the baseline sCr as a potential predictor, but the result was unsatisfactory. The sCr was not an independent predictor for predicting the occurrence of CRS-1 in ADHF, and its AUROC was only 0.68. Moreover, the level of sCr in AKI does not rise significantly until 72 h after the initial insult to the kidney [26]. Therefore, baseline creatinine of most patients with ADHF may be normal at admission. Thus, as the diagnostic criterion of AKI or CRS-1, creatinine has not been discussed as a predictor of AKI or CRS-1 in previous studies.

In addition, Hahn et al. reported that uNT-proBNP concentrations need to be corrected for uCr excretion to reduce bias between subjects [21]. Chen et al. found that uNT-proBNP/uCr can better predict future emergency department visits of patients with HF than uNT-proBNP and blood NT-proBNP [22]. However, no study far has yet shown that uNT-proBNP/uCr is a better predictor than uNT-proBNP for CRS-1 onset and outcomes in ADHF patients. Our results supported that the independent predictive power of the uNT-proBNP/uCr ratio for the occurrence of CRS-1 was significantly higher than that of uNT-proBNP and pNT-proBNP in patients with ADHF. The same was true for predicting clinical outcomes. This is in line with the earlier study [22]. The reason why uNT-proBNP was better than pNT-proBNP is still unknown. We speculate that this may be related to the clearance of NT-proBNP [34], leading to the possibility that the diagnostic performance of uNT-proBNP assays may be assay-specific [35]. A previous study suggested that uNT-proBNP may be more stable than pNT-proBNP for evaluating renal function in AHF patients [36]. Confusingly, the multivariate Cox regression revealed that the uNT-proBNP/uCr ratio was negatively influenced the clinical outcome, which requires further study on the reasons. So far, the ideal marker for early detection of CRS-1 has remained elusive. Compared with pNGAL, both the newly found pPENK and the noninvasive test of uNT-probNP/uCr may be two more promising novel biomarkers for early predicting CRS-1 occurrence and vulnerable-phase outcome in patients with ADHF.

## Limitations

This study has several limitations. First, our sample size is relatively small in two centers. Large-scale multicenter research is needed to validate the newly discovered conclusion. Second, we could not get the data about patient with AKI at admission or pre-admission, which may affect the integrity of the study. Third, the follow-up duration was short for the primary endpoint, necessitating additional follow-up together more detailed information for long-term results. Forth, the tested urine samples are frozen rather than fresh, which may have affected the results. Besides, previous studies have shown that therapeutic agents, such as beta blocker [37], were associated with worsening renal function in AHF, but we did not find similar results, which needs further investigation.

## Conclusions

This study determined the predictive value of pPENK (pg/mL) and uNT-proBNP/uCr ratio (pg/nmol) for early diagnosis of CRS-1 and vulnerable-phase prognosis in ADHF patients. They may become two novel biomarkers for the early prediction of CRS-1 in patient with ADHF. Meanwhile, they may play an important role in evaluating the composite of HF readmission or all-cause death 90 d after discharge in ADHF patients. The newly found pPENK and the noninvasive uNT-proBNP/uCr ratio may be useful tools in clinical settings for early detection of CRS-1 and prognosis assessment in ADHF patients in the future.

## Abbreviations

ADHF: acute decompensated heart failure
AKI: acute kidney injury
AUROC: area under the receiver operating characteristic curve
BNP: B-type natriuretic peptide
CKD: chronic kidney disease
CRS-1: cardiorenal syndrome type 1
KDIGO: Kidney Disease Improving Global Outcomes
NGAL: neutrophil gelatinase-associated lipocalin
NT-proBNP: amino-terminal pro-B-type natriuretic peptide
PENK: proenkephalin
sCr: serum creatinine
